# Supplementary material for: Multi-neuron connection using multi-terminal floating–gate memristor for unsupervised learning
Source: Nat Commun. 2023 May 27;14:3070. doi: 10.1038/s41467-023-38667-3 (PMC10224934; doi:10.1038/s41467-023-38667-3)
Supplement: Supplementary file 1 — Supplementary Information [file 41467_2023_38667_MOESM1_ESM.pdf]

Supplementary Information

Multi-terminal floating–gate memristor based artificial neuron for spiking neurosynaptic networks

Ui Yeon Won[1,2,†], Quoc An Vu[3,†], Sung Bum Park[1,†], Mi Hyang Park[1,†], Van Dam Do[1], Hyun Jun Park[4], Heejun Yang[5], Young Hee Lee[3,6,*], Woo Jong Yu[1,*]

Affiliations

[1]Department of Electrical and Computer Engineering, Sungkyunkwan University, Suwon 16419, Republic of Korea.

[2]Hyundai motors group, Electronic Devices research Team, Uiwang, 16082, Republic of Korea

[3]IBS Center for Integrated Nanostructure Physics, Institute for Basic Science, Sungkyunkwan University, Suwon 16419, Republic of Korea.

[4]Display R&D Group, Mobile Communication Business, Samsung Electronics, Suwon 16677, Korea

[5]Department of Physics, Korea Advanced Institute of Science and Technology, Daejeon 34141, Korea.

[6]Department of Energy Science, Sungkyunkwan University, Suwon 16419, Republic of Korea.

[†]These authors contributed equally to this work.

*Corresponding author: leeyoung@skku.edu (Y. H. Lee), micco21@skku.edu (W. J. Yu)

Structure	Polycrystalline MoS_2	Li+ migration MoS_2	FGMEM
Number of terminals	6	5	5
On/off ratio (max)	10^4	10^2	10^8
On/off ratio at 1,000 s retention	10	4	10^6
Non-linearity potentiation	4	6	0
Application	Hetero-synaptic	Hetero-synaptic	**Multi-neuron connnection**
Reference	[29]	[30]	Our work

Table S1. Comparison of memristors and memtransistors.

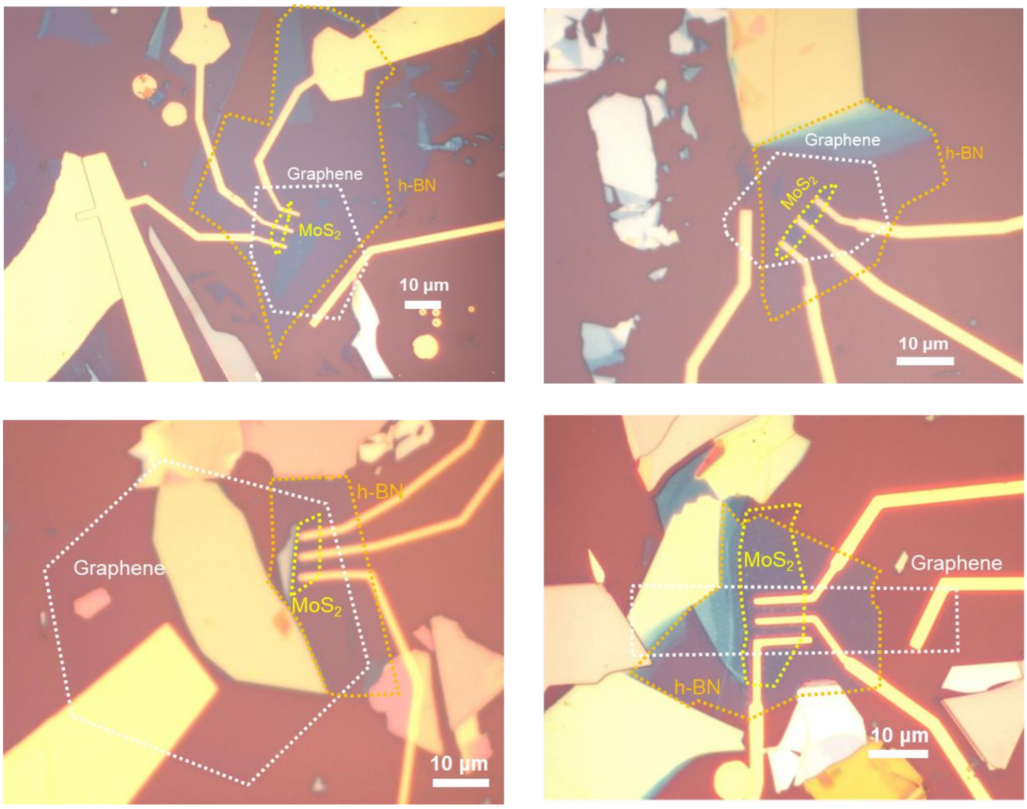

Supplementary Figure S1. Optical images of various multiterminal MT-FGMEMs formed of MoS_2/h-BN/graphene heterostructures.

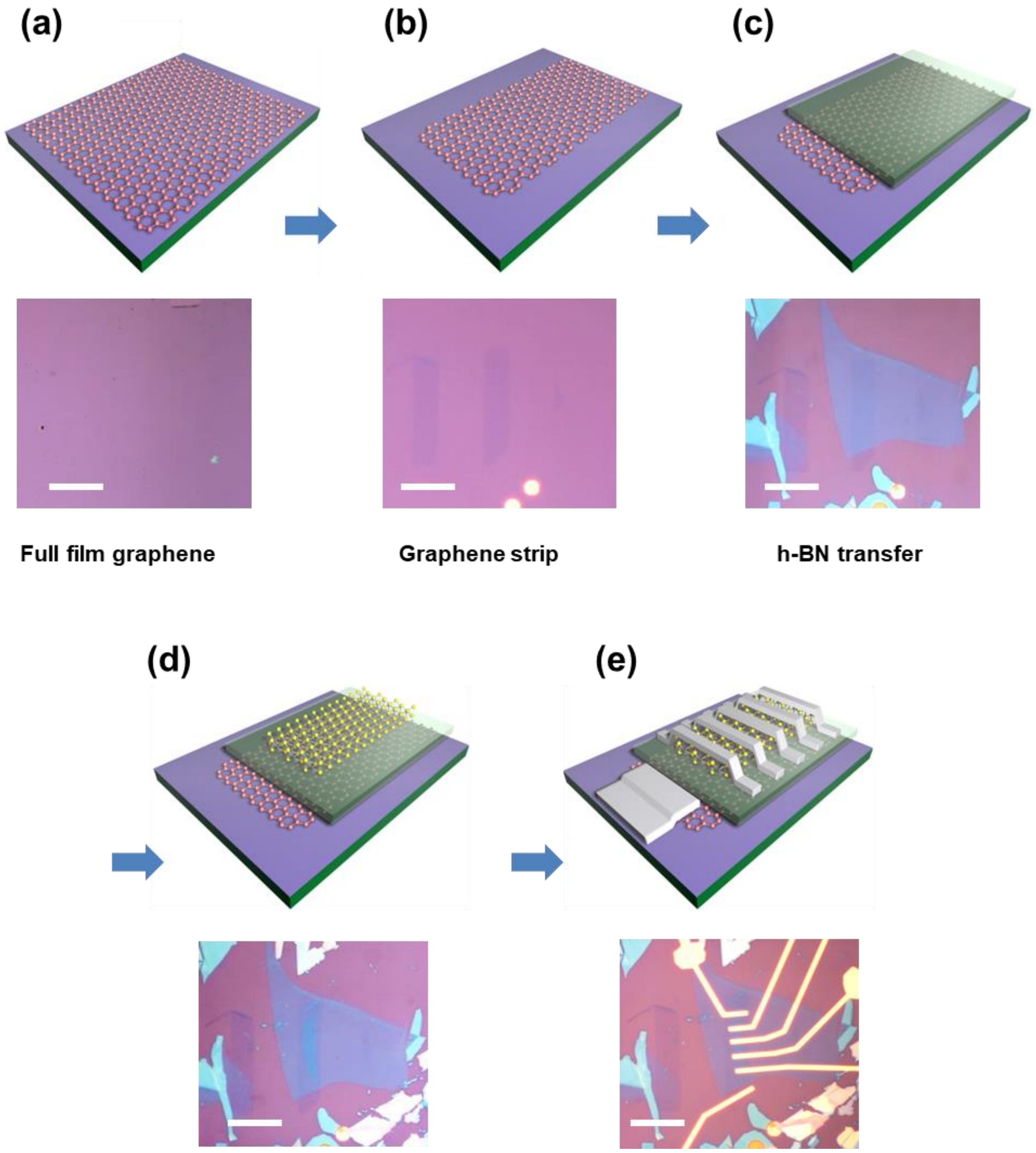

Supplementary Figure S2. Fabrication procedure of MoS_2/h-BN/graphene heterostructures. Stepwise fabrication of MoS_2/h-BN/graphene stacks illustrated with schematics (top panel) and optical microscope images (bottom panel). **a**, Large-area CVD-grown monolayer graphene was transferred onto a 300-nm SiO_2-covered Si substrate. **b**, Graphene was patterned into 10 × 50 μm strips. **c** and **d**, Sequential transfer of h-BN and MoS_2 onto the graphene strip using a dry transfer technique. **e**, Patterning and deposition of Cr/Au (10/50 nm) electrodes using e-beam lithography and e-beam/thermal evaporation. All scale bars are 20 μm.

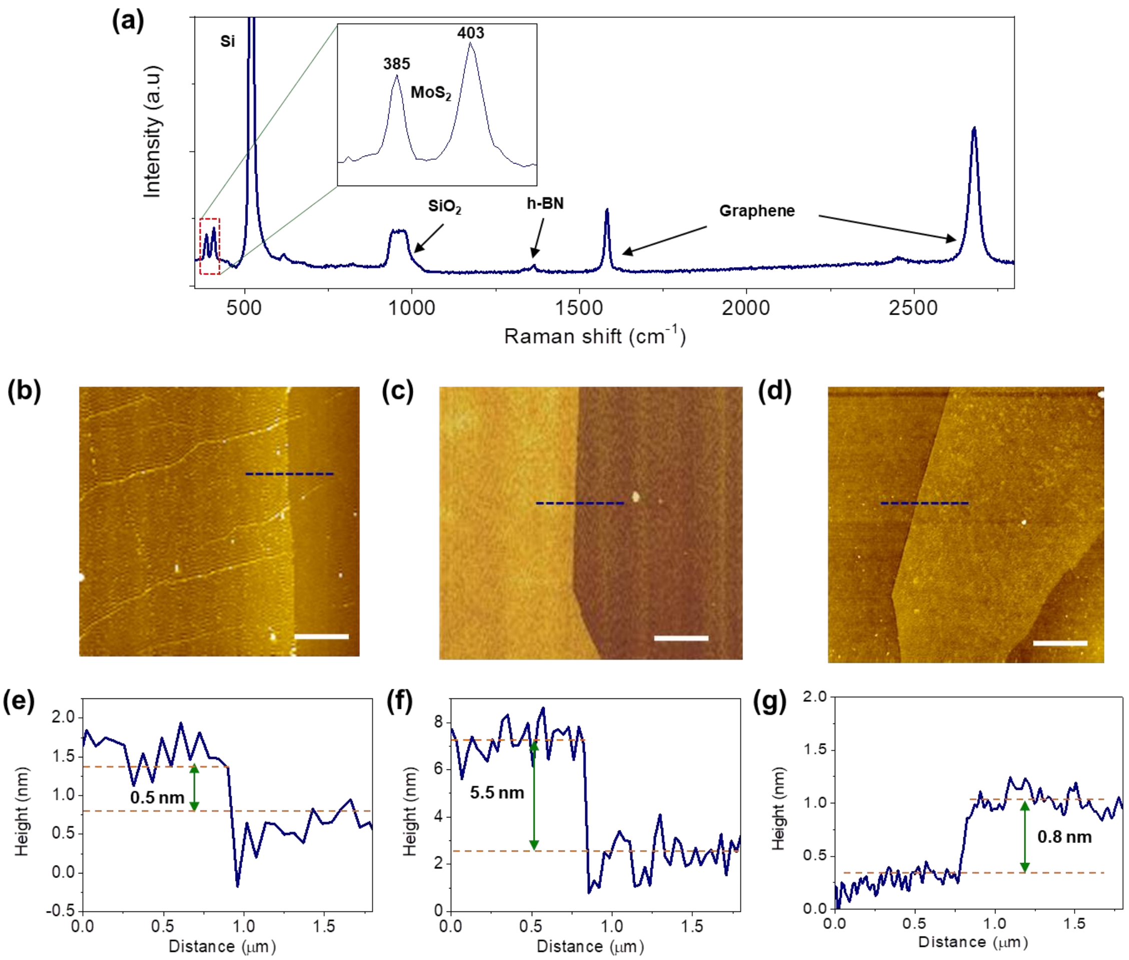

Supplementary Figure S3. Raman spectrum and AFM analysis of MoS_2/h-BN/graphene heterostructure. a, The Raman spectrum taken at an overlapping area involves the identification of Raman peaks of MoS_2, h-BN, and graphene, respectively. The inset shows the E^1_{2g} and A_{1g} peaks at 383 cm^{-1} and 403 cm^{-1}, indicating the monolayer structure of MoS_2. **b-d**, AFM scanning images (top) and **e-g** height profiles (bottom) of CVD-grown graphene (**b**), multilayer h-BN (**c**), and monolayer MoS_2 (**d**). All scale bars are 1 μm.

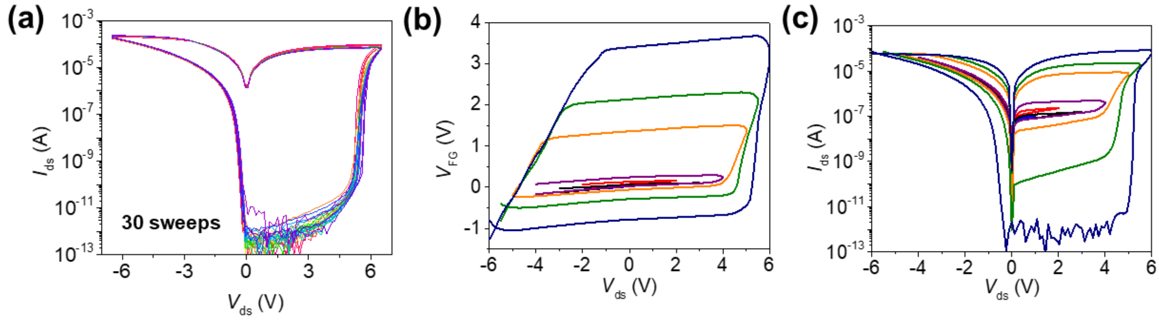

Supplementary Figure S4. Modulation of graphene floating-gate potential and MoS_2 channel current under source–drain bias. a, Typical memory behavior in 30-sweeps two-terminal measurement of MT-FGMEM. Our MT-FGMEM demonstrates highly reliable memory behavior with negligible curve change in 30 times voltage sweeps. **b-c,** The potential hysteresis of the graphene floating gate (**b**) and current hysteresis of the MoS_2channel (**c**) under source–drain V_{12} sweeps with different amplitudes from ±1 V to ±6 V. The floating-gate potential and current were measured simultaneously by using a Keithley 4200 source/measure unit. At the voltage sweeps of less than ±4 V, we observe no potential change in FG and no corresponding memory window. At the voltage sweeps higher than ±5 V, a clear potential change in FG and the corresponding memory window are observed. Evidently, our MT-FGMEM works by charging and discharging in graphene FG.

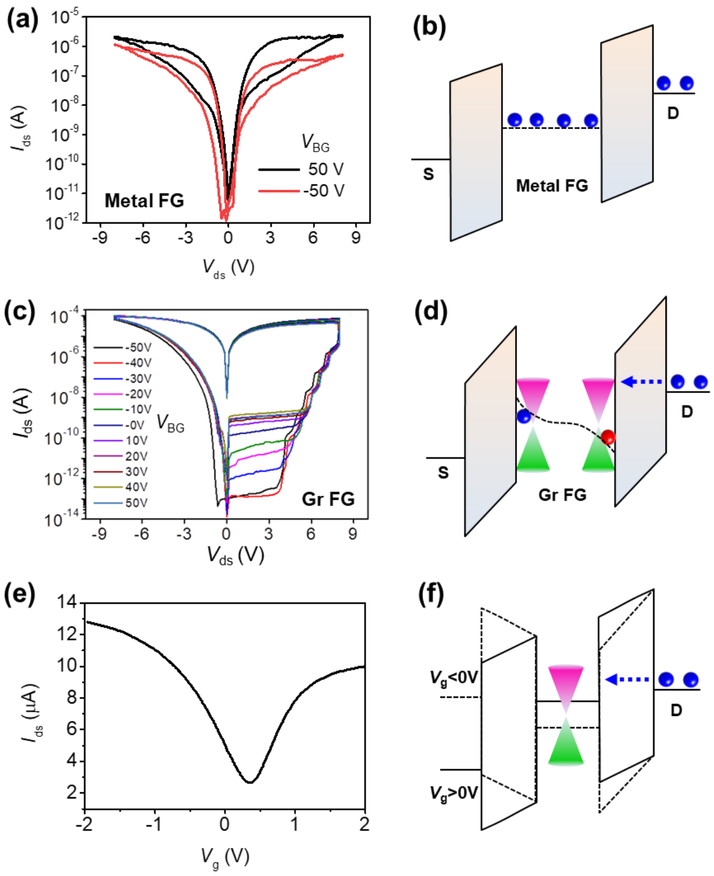

Supplementary Figure S5. Memory behavior of metal FGMEM. a, Memtransistor behavior of FGMEM with metal-FG under back gate voltage (V_{BG}) on Si/SiO_2 substrate. **b,** Energy band diagram of drain/insulator/metal FG/insulator/source under the erasing drain bias. **c,** Memtransistor behavior of FGMEM with graphene-FG under back gate voltage (V_{BG}) on Si/SiO_2 substrate. **d,** Energy band diagram of drain/insulator/graphene FG/insulator/source under the erasing drain bias. **e,** Transfer curve of our graphene/h-BN/metal heterostructure. Our graphene shows clear E_F shift along the gate voltage with Drac-point at 0.2 V. **f,** Energy band diagram of gate/insulator/graphene FG/insulator/drain under positive V_g (solid line) and negative V_g (dashed line). The on/off current ratio change by gate voltage is related to graphene E_F shift. At negative gate voltage (V_g < 0 V), positive holes are attracted to graphene layer, shifting EF downward. Then the band bending of tunneling insulator becomes steeper, resulting in more electron tunneling. At the positive gate voltage (V_g > 0 V), negative electrons are attracted to graphene layer, shifting E_F upward. Then the band bending of tunneling insulator becomes smoother, resulting in less electron tunneling.

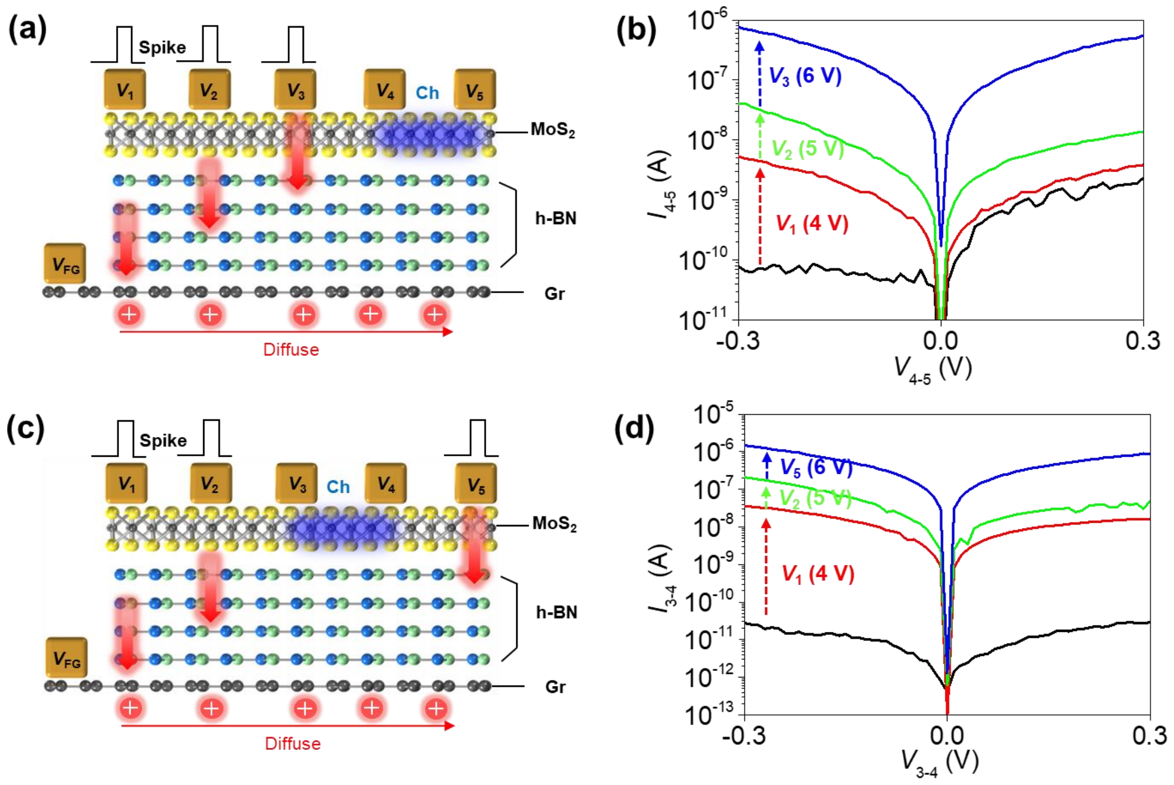

Supplementary Figure S6. a-b, The schematics and current change of V_4–V_5 channel by V_1 = 4 V, V_2 = 5 V and V_3 = 6 V spikes and **c-d,** V_3-V_4 channel by V_1 = 4 V, V_2 = 5 V and V_5 = 6 V spikes. Both results show similar current changes because all MoS_2 channels are modulated by sharing the graphene floating gate.

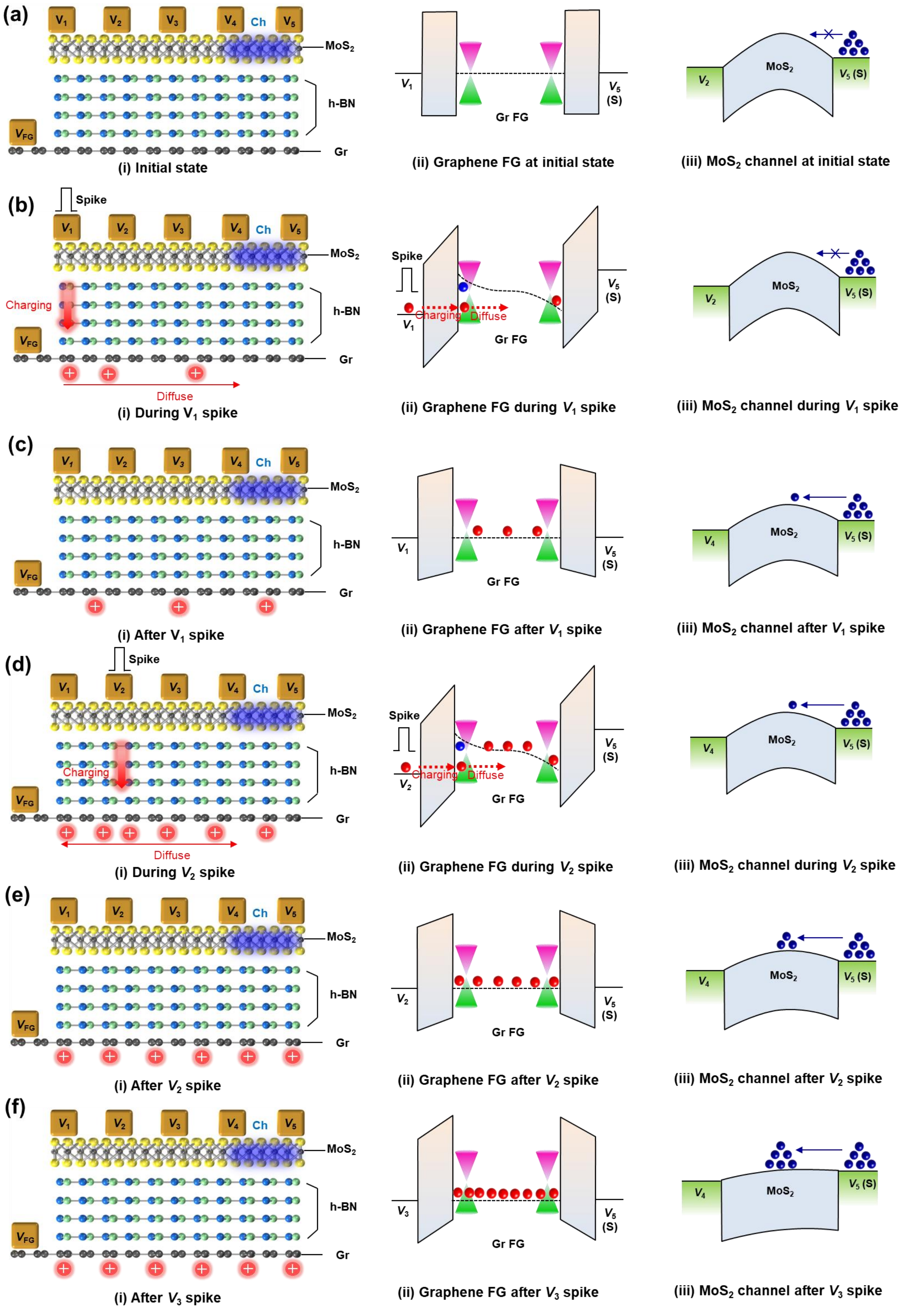

Supplementary Figure S7. Schematic energy band diagrams of V_1~V_3/h-BN/graphene FG/h-BN/V_5(S) and V_4/MoS_2/V_5(S) at different charging scenarios of V_1, V_2 and V_3 spikes. (a)

Initial turn-off state. (b) During V_1 spike biasing. (c) After V_1 spike. (d) During V_2 spike biasing. (e) After V_2 spike. (f) After V_3 spike.

At initial state (Figure S7a), the MoS_2 channel is turned-off. During the V_1 spike biasing (Figure S7b), local positive spike attracts the electron in graphene, shifting the Fermi level (E_F) of graphene. It enhances band bending of h-BN and hole tunneling through h-BN. Tunneled holes diffuse through graphene layer. After the V_1 spike (Figure S7c), trapped holes evenly distribute through graphene layer and shift MoS_2 conduction barrier downward. During the next V_2 spike biasing (Figure S7d), holes tunnel to graphene FG as same mechanism as Figure S7b. After the V_2 spike (Figure S7e), more holes are evenly trapped in graphene layer and shift MoS_2 conduction barrier further downward. Next V_3 spike also further increase the number of holes in graphene FG and further decrease the MoS_2 conduction barrier (Figure S7f).

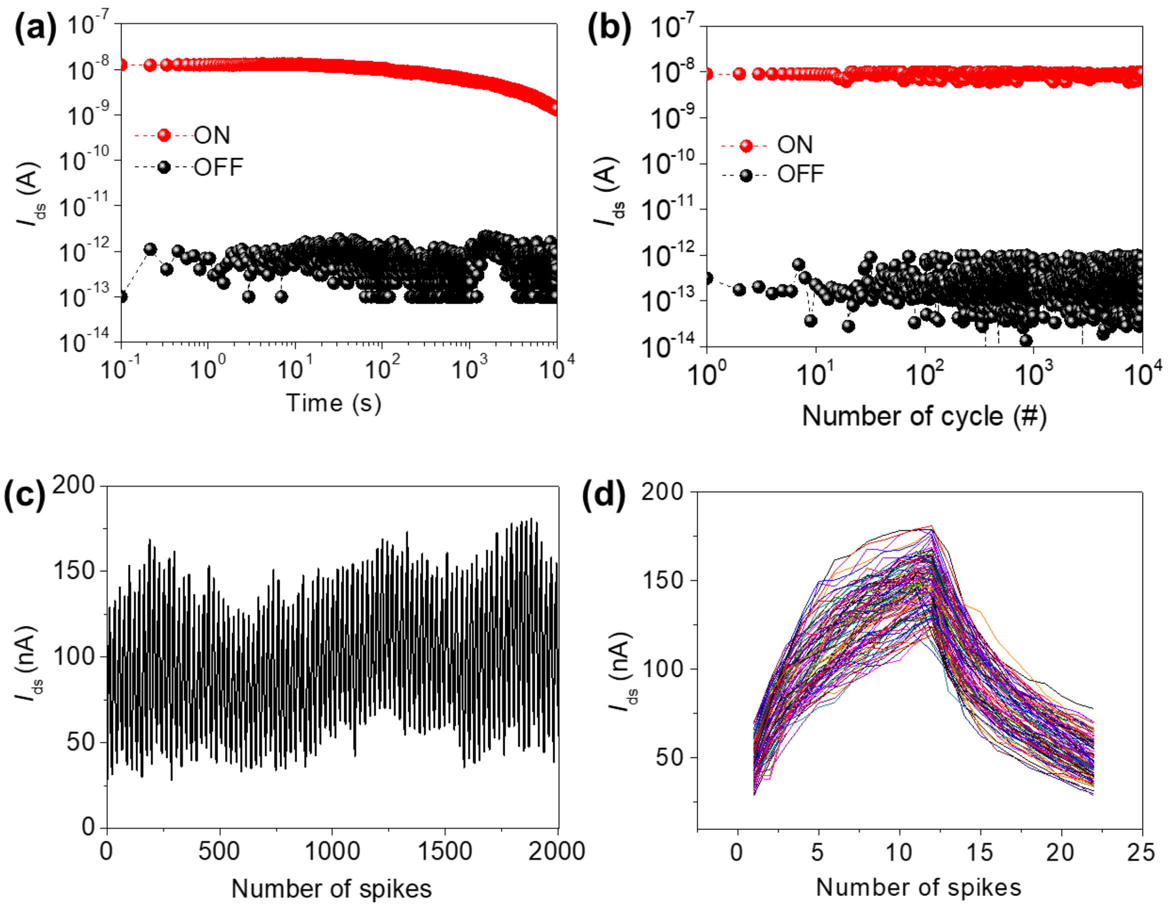

Supplementary Figure S8. Memory characteristics of FGMEM. a, 2-level retention for 10,000 s and **b,** endurance for 10,000 cycles with set (6 V) and reset (-6 V) spike. **c-d,** Multi-level endurance for 2000 spikes.

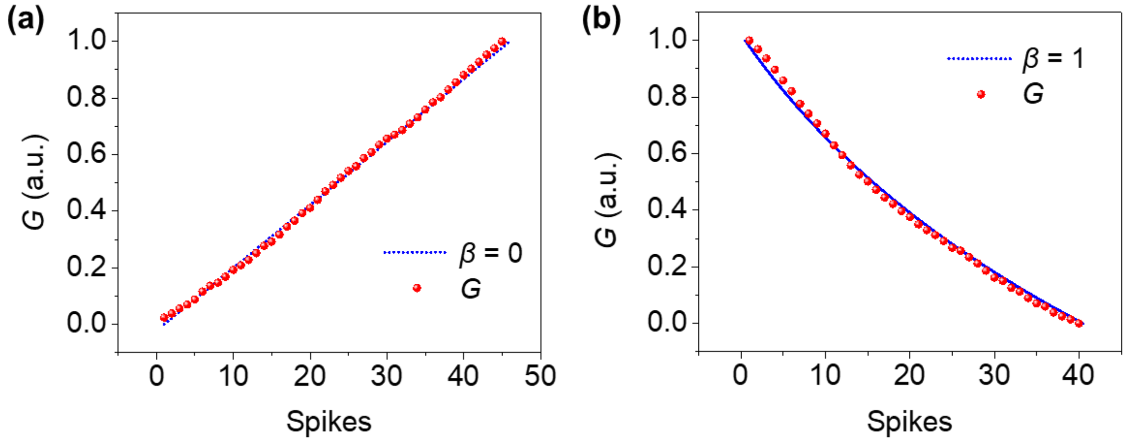

Supplementary Figure S9. Linearity of MT-FGMEM conductance change along sequential spikes. a-b, The potentiation and depression of MT-FGMEM. Red circles are the conductance change of MT-FGMEM along the sequential spikes. The blue dotted line indicates the fitting line obtained by the equation $\Delta G = \alpha e^{-\beta \frac{G - G_{\min}}{G_{\max} - G_{\min}}}$.

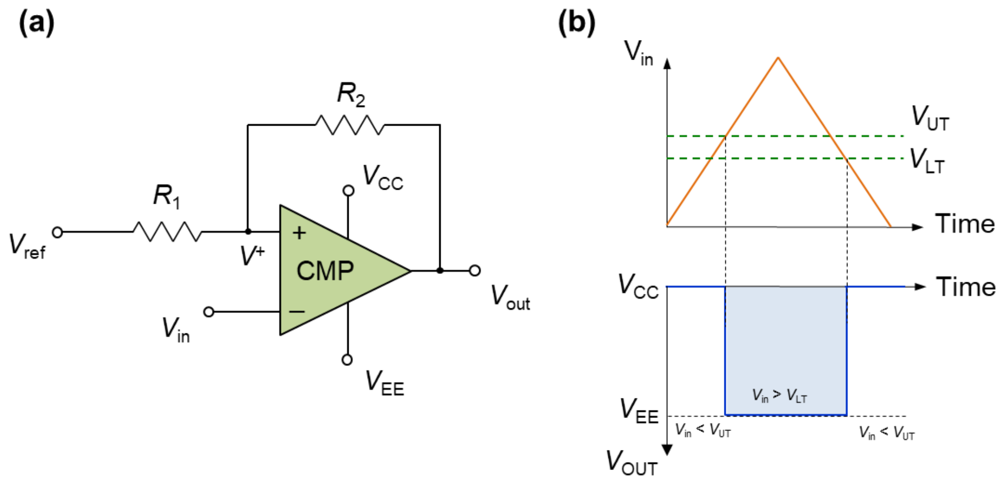

Supplementary Figure S10. Characteristic of a comparator with the Schmitt trigger setup. a-b, Schematic (**a**) and output characteristic (**b**) of the comparator.

Schmitt trigger setup. To stabilize the resting potential after spikes were generated by the comparator, a Schmitt trigger containing a comparator and resistors can be implemented to create a positive feedback loop. The comparator compares the difference between the input and reference voltages (threshold voltage). Depending on whether the input is lower or higher than the reference, the output voltage is changed. The Schmitt trigger has two thresholds (upper threshold (UT) and lower threshold (LT)) depending on its present output. If the output is high, it will change when the input is higher than the UT. In contrast, if the output is low, it will change when the input is lower than LT. According to Kirchhoff's current law,

$$\frac{V^+ - V_{\mathrm{ref}}}{R_1} + \frac{V^+ - V_{\mathrm{out}}}{R_2} = 0 \quad (1)$$

$$V^+ = \frac{R_2}{R_1+R_2} V_{\mathrm{ref}} + \frac{R_1}{R_1+R_2} V_{\mathrm{out}} \quad (2)$$

If we assume that the output voltage is V_{EE},

$$V^+ = \frac{R_2}{R_1+R_2} V_{\mathrm{ref}} + \frac{R_1}{R_1+R_2} V_{\mathrm{EE}} \quad (3)$$

$$V_{in} - V^+ < 0 \quad (4)$$

Then,

$$V_{\mathrm{in}} < \frac{R_2}{R_1+R_2} V_{\mathrm{ref}} + \frac{R_1}{R_1+R_2} V_{\mathrm{EE}} \quad (5)$$

Therefore,

$$V_{\mathrm{LT}} = \frac{R_2}{R_1+R_2} V_{\mathrm{ref}} + \frac{R_1}{R_1+R_2} V_{\mathrm{EE}} \quad (6)$$

If we assume that the output voltage is V_{CC},

$$V^{+} = \frac{R_2}{R_1+R_2} V_{\mathrm{ref}} + \frac{R_1}{R_1+R_2} V_{\mathrm{CC}} \quad (7)$$

$$V_{\mathrm{in}} - V^{+} > 0 \quad (8)$$

Then,

$$V_{\mathrm{in}} > \frac{R_2}{R_1+R_2} V_{\mathrm{ref}} + \frac{R_1}{R_1+R_2} V_{\mathrm{CC}} \quad (9)$$

Therefore,

$$V_{\mathrm{UT}} = \frac{R_2}{R_1+R_2} V_{\mathrm{ref}} + \frac{R_1}{R_1+R_2} V_{\mathrm{CC}} \quad (10)$$

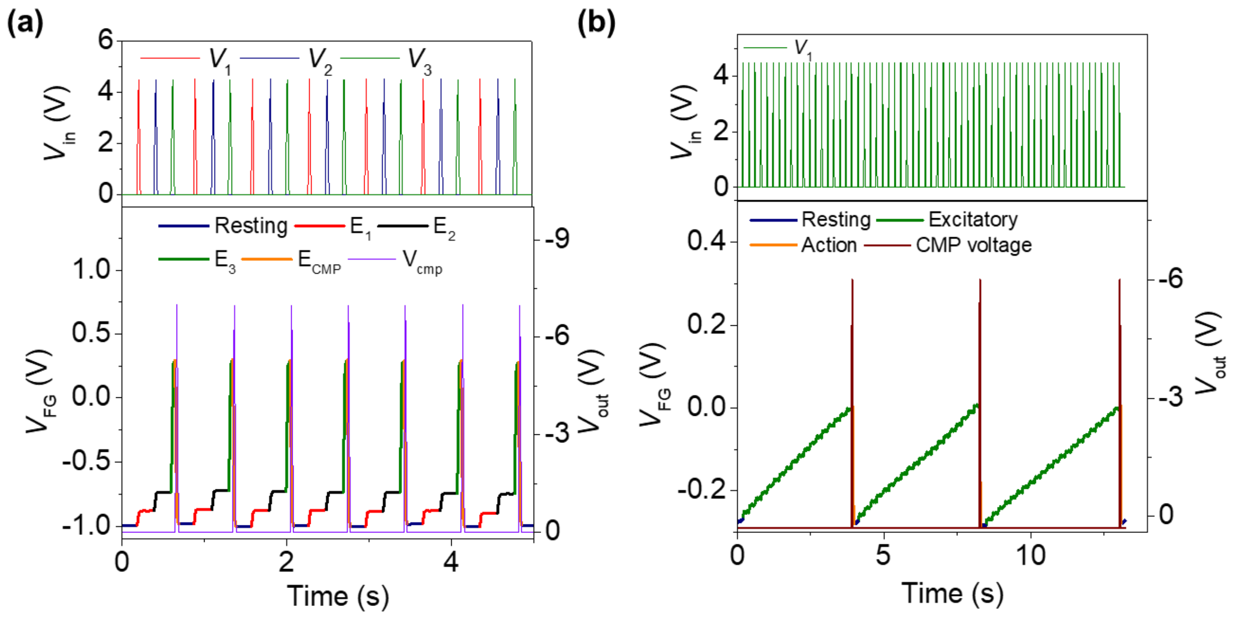

Supplementary Figure S11. Other artificial neurons configured by the FG and comparator. a, Neuronal LIF with three potentiation pre-neurons (V_1, V_2, and V_3). The spike height and width are 4.5 V and 0.1 s, respectively. **b,** Neuronal LIF with single potentiation pre-neuron (V_1). The spike height and width are 4.2 V and 1 ms.

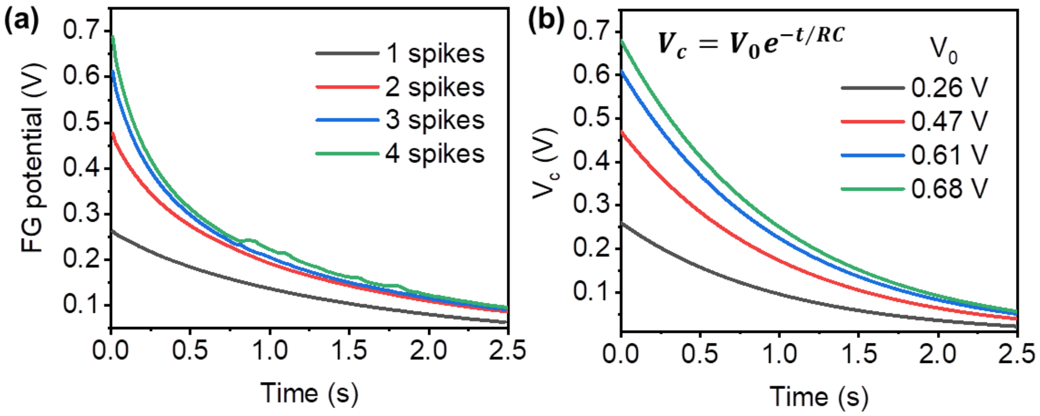

Supplementary Figure S12. a, The leaky profile of floating gate in Figure 3i. **b**, Simulation of capacitor discharge: $V_c = V_0 e^{-t/RC}$, where V_c is the capacitor voltage, V_0 is the initial capacitor voltage, t is the time, R is the resistance (resistance of tunneling layer), and C is the capacitance (capacitance of our FG).

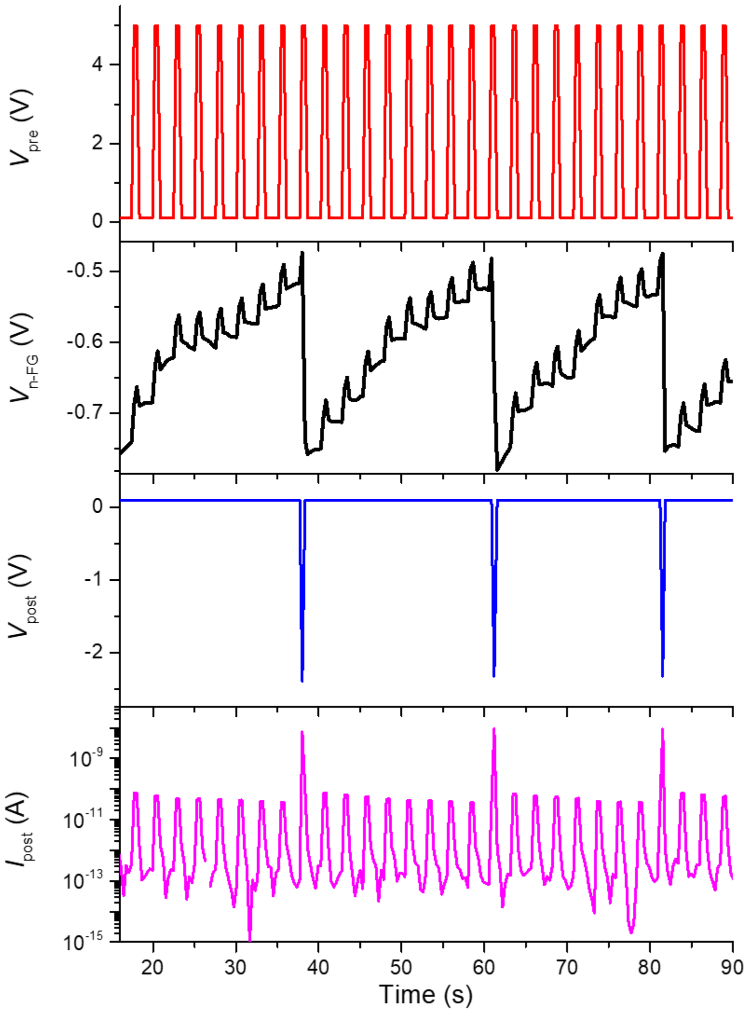

Supplementary Figure S13. Current consumption of neuron (FG-com). The energy consumption is calculated by $\Delta(V_{pre} - V_{post}) \times t_W \times I_{post}$.

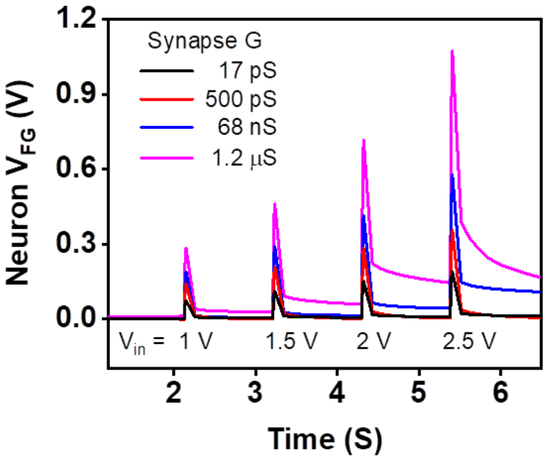

Supplementary Figure S14. Potential change of neuronal V_{FG} (membrane) by input spikes at different synaptic weight - conductance of MoS_2 (*Weighting*).

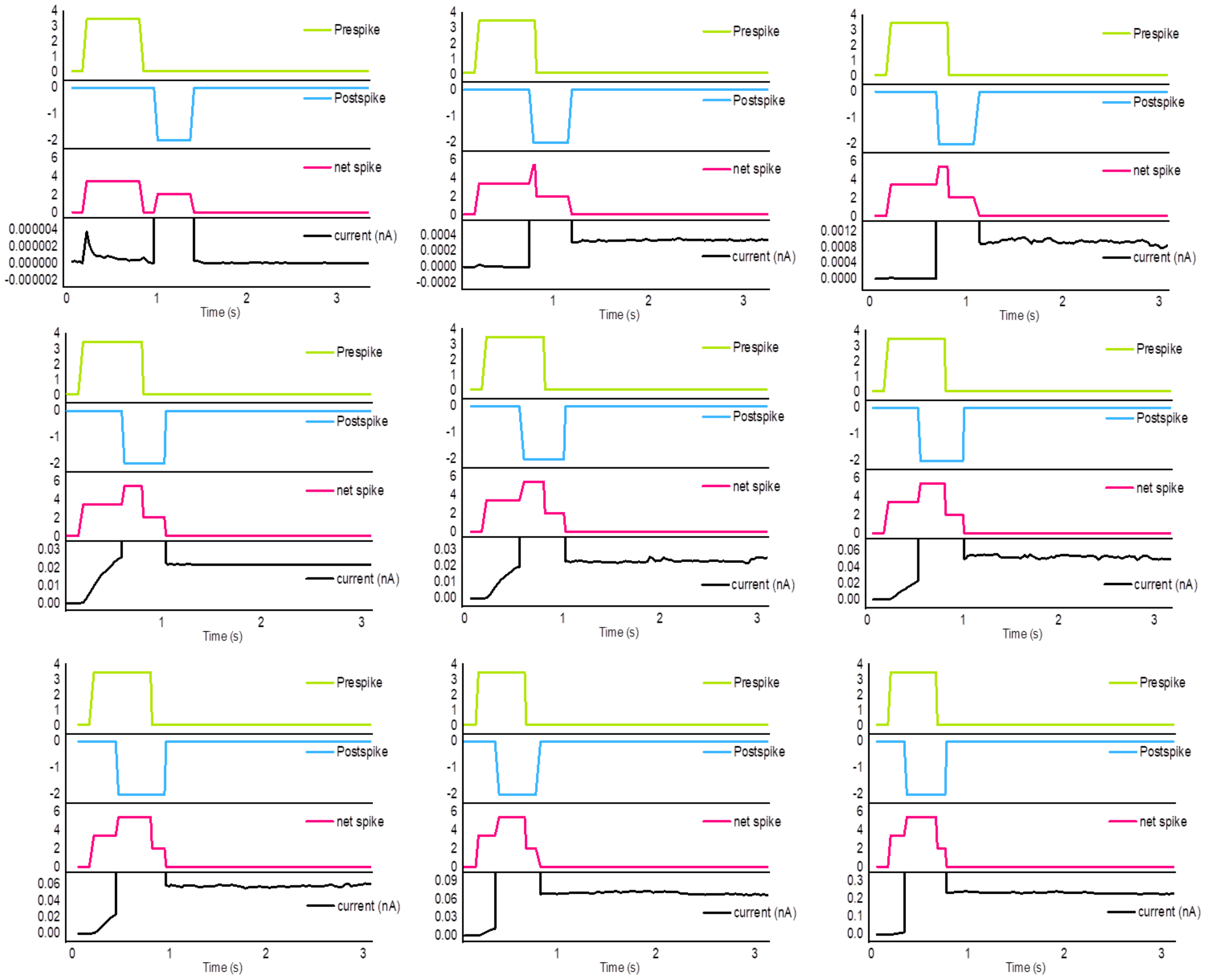

Supplementary Figure S15. Spike-timing-dependent plasticity of MT-FGMEM.

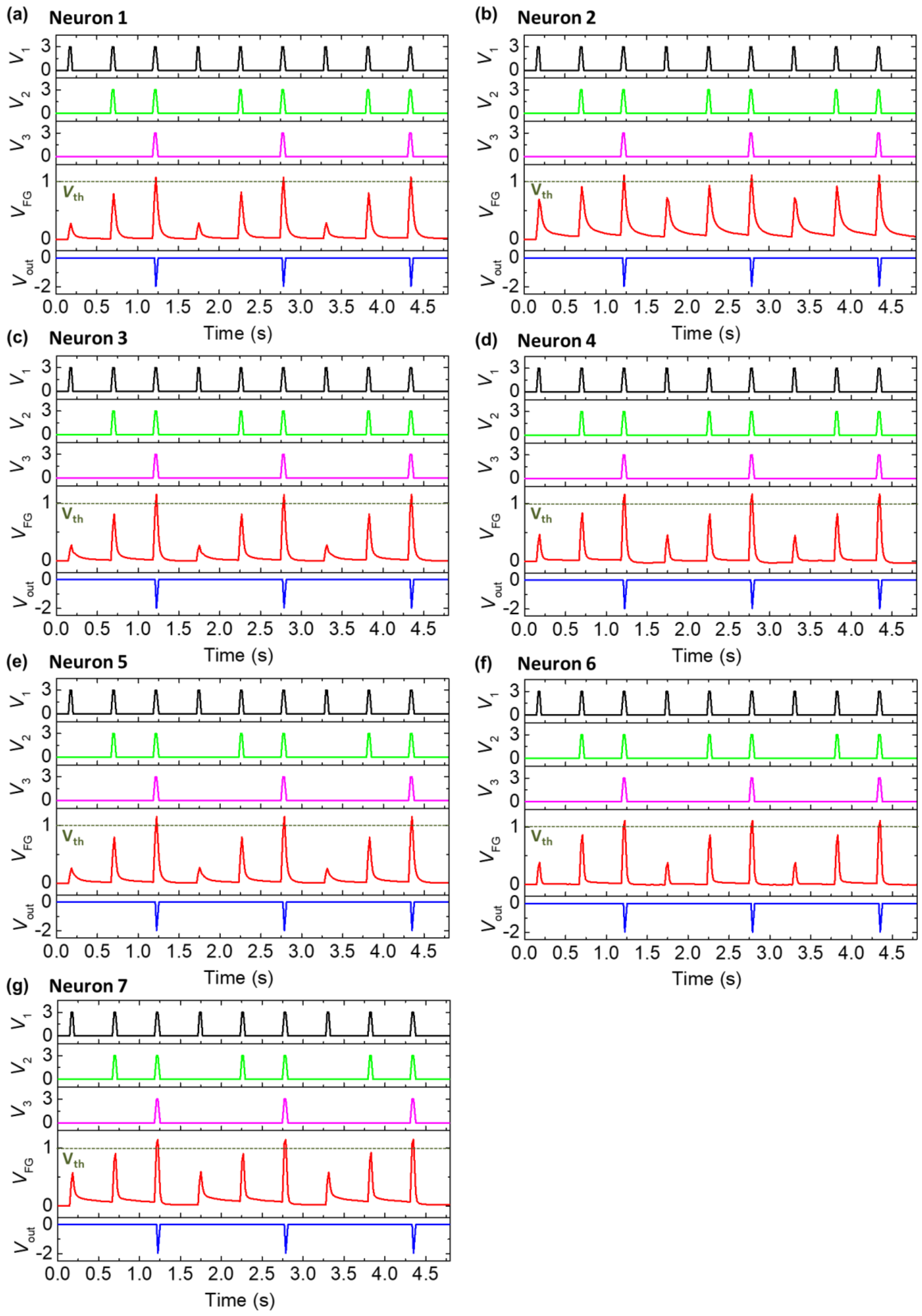

Supplementary Figure S16. Spatial summation of artificial neurons in neurosynaptic network (Fig. 5a).

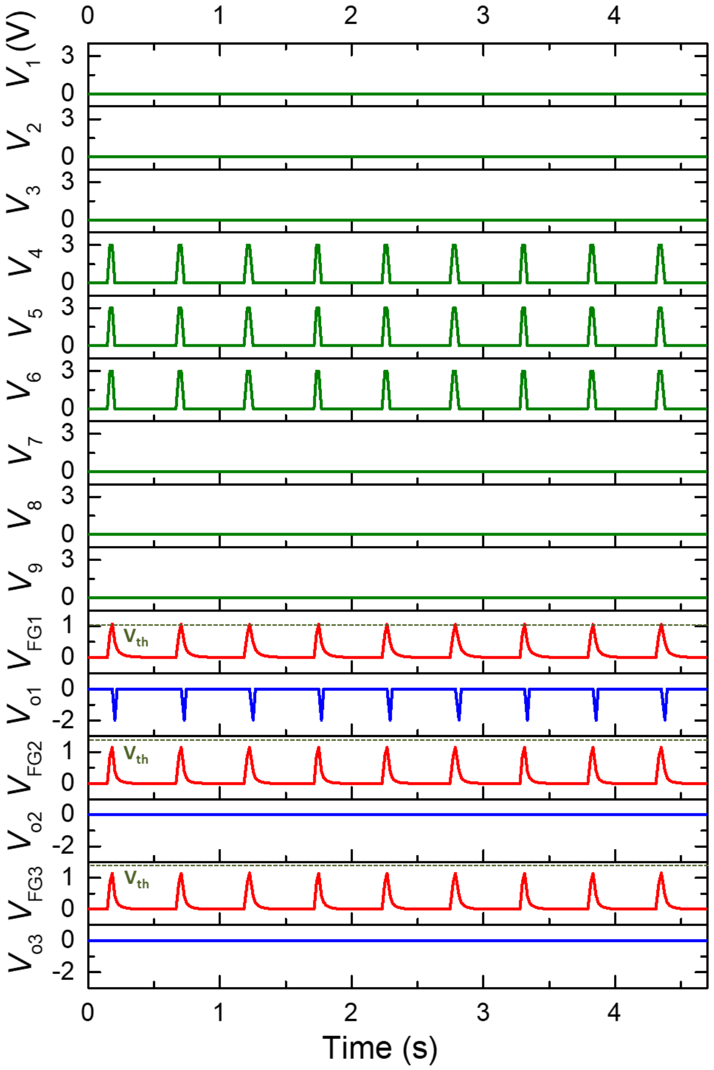

Supplementary Figure S17. Potentials of input spikes ($V_{1\text{-}9}$), membrane potentials of N1-3 ($V_{FG1\text{-}3}$) and output spikes of N1-3 ($V_{o1\text{-}3}$) during training vertical line '|' on N1 neuron.

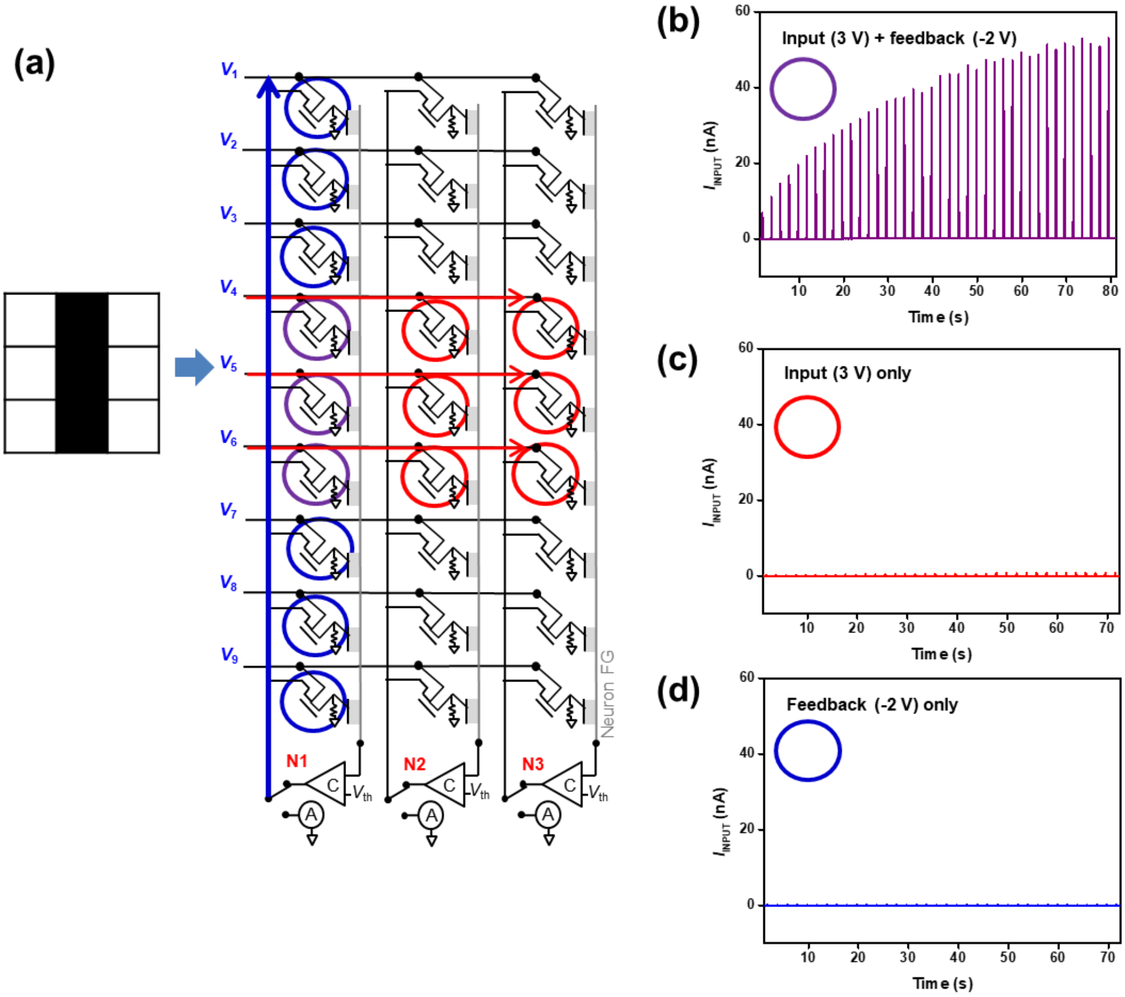

Supplementary Figure S18. Current change of synapse at various spike combinations. a, Input (V_{pre}) and feedback (V_{post}) spike application to the synapses at '|' training. **b**, Current change (Weight update) by overlapped input (3 V) and feedback (-2 V) spikes (in purple circles). **c**, No current change (no weight update) by input spikes only (in red circles). **d**, No current change (no weight update) by feedback spikes only (in blue circles).

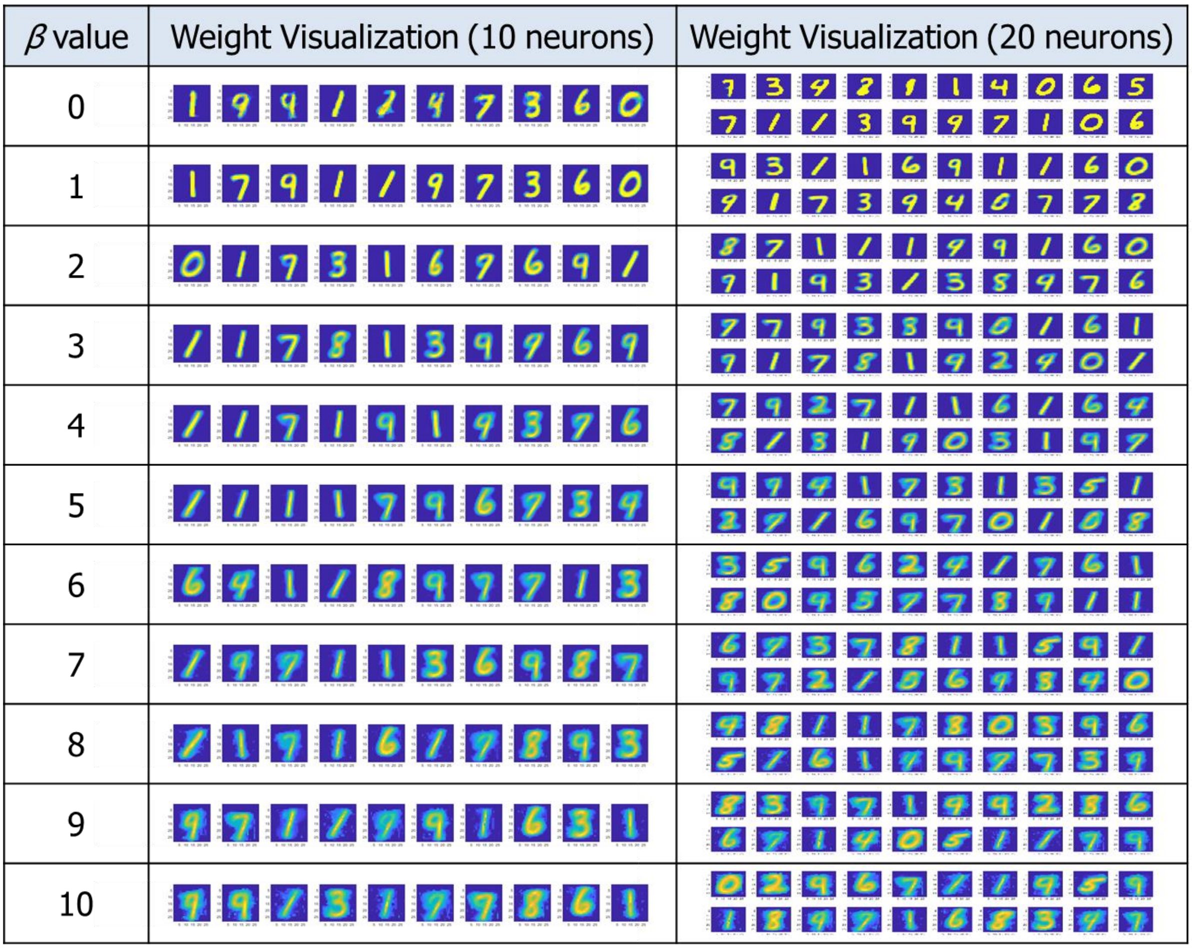

Supplementary Figure S19. Visualized synaptic weights after unsupervised learning. Rows and columns present the nonlinearity factor (β) and number of post-neurons (1st column for 10 post-neurons and 2nd column for 20 post-neurons), respectively. At the ideal linearity (β = 0), the boundary of the visualized digit is very clear, allowing an accurate classification of the MNIST test digits. However, the increasing nonlinearity blurs the boundaries of the visualized digit, resulting in an inaccurate classification of the ambiguous MNIST test digits.

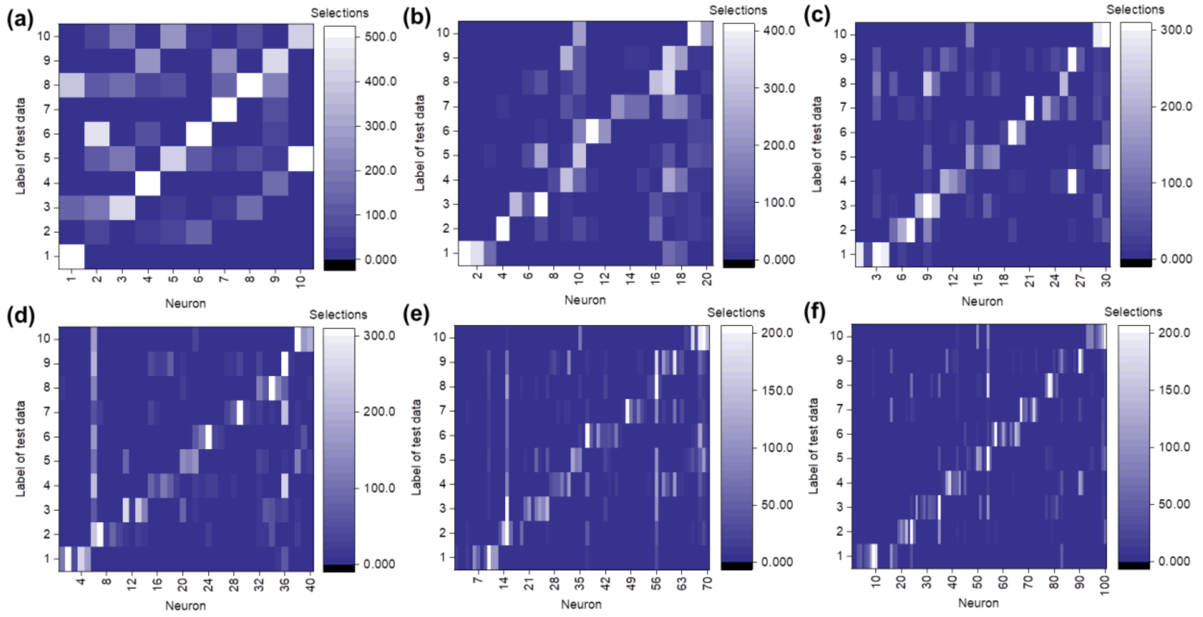

Supplementary Figure S20. Number of neuron selections by (**a**) 10, (**b**) 20, (**c**) 30, (**d**) 40, (**e**) 70, and (**f**) 100 post-neurons for the test label.
